# A novel lncRNA, TCONS_00006195, represses hepatocellular carcinoma progression by inhibiting enzymatic activity of ENO1

**DOI:** 10.1038/s41419-018-1231-4

**Published:** 2018-12-05

**Authors:** Songman Yu, Ning Li, Zebing Huang, Ruochan Chen, Panpan Yi, Rui Kang, Daolin Tang, Xingwang Hu, Xuegong Fan

**Affiliations:** 1 Department of Infectious Diseases, Hunan Key Laboratory of Viral Hepatitis, Xiangya Hospital, Central South University, Changsha, China; 2 Department of Blood Transfusion, Xiangya Hospital, Central South University, Changsha, China; 3 Department of Surgery, University of Pittsburgh, Pittsburgh, PA USA

## Abstract

Hepatocellular carcinoma (HCC) is one of the most common malignancies and has an unfavorable prognosis. The hepatitis B virus X (HBx) protein has been reported to be closely associated with hepatocarcinogenesis. Meanwhile, emerging evidence has indicated that long noncoding RNAs (lncRNAs) are involved in the pathogenesis and progression of cancers. Our previous investigation has demonstrated that HBx could promote HCC by regulating the expression levels of various lncRNAs. In this study, we identified an lncRNA, lncRNA-TCONS_00006195 (termed lncRNA-6195), which was downregulated in HBV-related HCC tissues compared with its expression in adjacent noncancerous hepatic tissues. Clinical data showed that a low level of lncRNA-6195 was correlated with a high Edmondson–Steiner grade of the tumor and a poor prognosis in HCC patients. Furthermore, lncRNA-6195 acted as a tumor repressor in the development of hepatitis B-related HCC, inhibiting HCC cell proliferation in vitro and in vivo. Moreover, lncRNA-6195 could combine with α-enolase (ENO1) and repress its enzymatic activity, thus further inhibiting the energy metabolism in HCC cells. Our results suggest that lncRNA-6195 represses the growth of HCC by inhibiting the enzymatic activity of ENO1. These findings provide new insights into the mechanisms underlying the lncRNA involvement in hepatocarcinogenesis and can serve as a basis for the development of novel strategies to hinder HCC.

## Introduction

Hepatocellular carcinoma (HCC) is one of the most common human malignancies and the third leading cause of cancer-related deaths worldwide^1^. Chronic hepatitis B virus (HBV) infection is the major cause of HCC in China. Although researchers have determined some factors contributing to HBV-induced HCC tumorigenesis, such as genomic instability, insertional mutagenesis, and epigenetic changes^2,3^, the underlying molecular mechanisms are still unclear.

Long noncoding RNAs (lncRNAs) are a class of transcripts that have more than 200 nucleotides and exhibit no protein-coding potential. Recently, emerging evidence has indicated that lncRNAs play critical roles in the pathogenesis and progression of cancers^4^. A number of lncRNAs, such as ATB (lncRNA activated by transforming growth factor-β)^5^, DANCR (differentiation-antagonizing non-protein-coding RNA)^6^, HEIH (lncRNA highly expressed in HCC)^7^, MVIH (lncRNA associated with microvascular invasion in HCC)^8^, and TP73-AS1 (P73 antisense RNA 1T)^9^, have been found to be dysregulated in and associated with HCC. These lncRNAs participate in various biological processes, including cell proliferation, apoptosis, invasion, and migration^10^. The HBV X (HBx) protein has been reported to be closely associated with HBV-induced hepatocarcinogenesis. In recent years, some lncRNAs, such as DREH^11^, UCA1^12^, and Unigene56159^13^, have been proven to be regulated by HBx and involved in the pathogenesis and progression of HBV-related HCC. However, the functions and mechanisms of most HBx-related lncRNAs in HCC are still unclear.

α-Enolase (ENO1) is an enolase isoform present in almost all adult tissues in mammals. It was originally characterized as a key enzyme of glycolysis, catalyzing the conversion of 2-phosphoglycerate (2PG) to phosphoenolpyruvate (PEP)^14^. After decades of research, scientists have demonstrated that besides its glycolytic function in normal processes, ENO1 also participates in several critical biological processes in cancer, including proliferation, migration, and invasion^15–18^.

In our previous study, we have used an lncRNA hybridization-based microarray and real-time polymerase chain reaction (PCR) to obtain the lncRNA expression profiles of L02/HBx and L02/pcDNA3.0 cell lines. In this study, we further investigated the biological function and the underlying mechanism of an HBx-upregulated lncRNA, lncRNA-TCONS_00006195 (termed lncRNA-6195), in vivo and in vitro to potentially find a new strategy to treat HCC.

## Results

### LncRNA-6195 is downregulated in HCC tissue

**Fig. 1:**
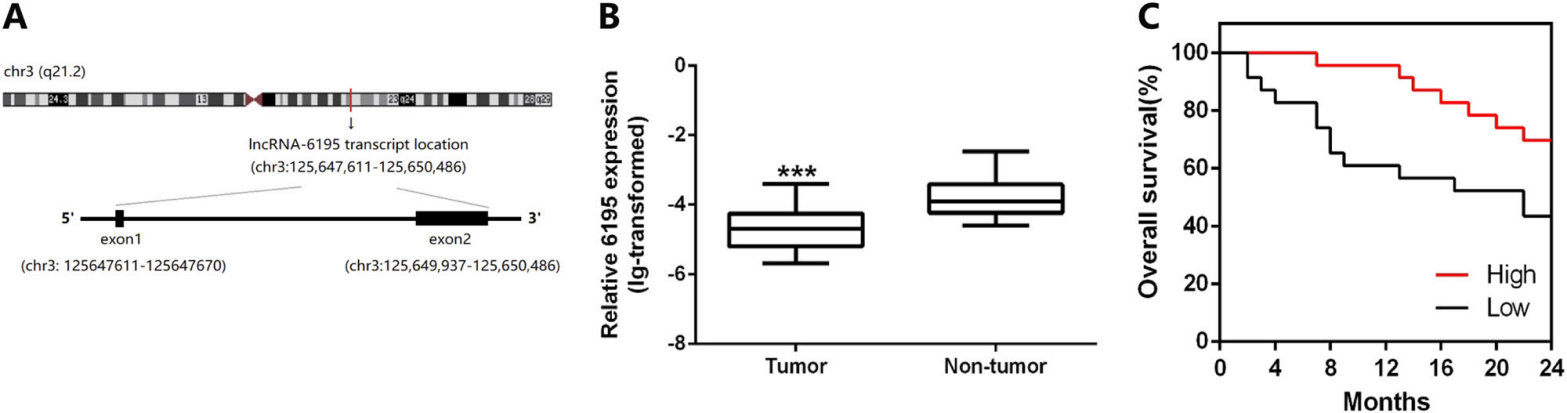
The lncRNA-6195 is downregulated in HCC and could be an independent prognostic factor to predict OS. **a** Transcript location of lncRNA-6195. **b** LncRNA-6195 is significantly downregulated in 47 HBV-related HCC patients’ HCC tissues compared with the corresponding adjacent non-tumorous tissues (***P < 0.001, paired-samples *t* test). **c** Kaplan–Meier analysis of OS based on lncRNA-6195 expression levels in 46 patients undergoing HBV-related HCC. The median expression level of lncRNA-6195 was used as the cutoff. Patients were divided into “High” group (whose lncRNA-6195 expression was higher than the median) and “Low” group (whose lncRNA-6195 expression was lower than the median). Compared with the high group, the OS (*P* = 0.042, log-rank test) were significantly lower in the low group

In our previous study^19,20^, we have found that compared with the control group, which was stably transfected with a blank plasmid (L02/pcDNA3.0), LO2/HBx cells had 323 upregulated and 421 downregulated lncRNAs (fold change >2.0, *P* < 0.05). Expression of lncRNA-6195 was one of the most significantly altered in LO2/HBx cells, and we speculated that lncRNA-6195 might play an important role in HCC. The lncRNA-6195-coding sequence, consisting of exons 1 and 2, is located on chromosome 3, between oxysterol-binding protein-related protein 11 (55,137 bp) at the 5′-end and translation initiation factor IF-2-like isoform X1 (105,706 bp) at the 3′-end (Fig. 1a). By searching for the lncRNA-6195 sequence in Lncipedia (https://lncipedia.org), we found that the PhyloCSF^21^ score of lncRNA-6195 was −83.9101, and the CPAT^22^ coding probability was 1.78%. The analysis result of PRIDE reprocessing 2.0^23^ was zero. Additionally, Lncipedia failed to predict any Bazzini small open reading frames^24^ or Lee translation initiation sites^25^ in lncRNA-6195. All these data suggest that the lncRNA-6195 transcript is consistent with an lncRNA.

To investigate the expression levels of lncRNA-6195 in HBV-related HCC, quantitative reverse transcription-PCR (qRT-PCR) was performed on 47 pairs of human HBV-related HCC tissues and matched non-tumorous liver tissues. The transcript levels of lncRNA-6195 were significantly downregulated in HCC tissues compared with those in adjacent non-tumorous hepatic tissues (*P* < 0.001; Fig. 1b). Additionally, the expression level of HBx in HCC tissue also significantly reduced compared with adjacent non-tumor tissues and the expression of lncRNA-6195 and HBx was positively correlated in HCC tissue (data not shown). Further, clinical data of these 47 patients showed that the low level of lncRNA-6195 was correlated with a high Edmondson–Steiner grade of the tumor (*P* < 0.05; Table 1). Kaplan–Meier analysis of 46 HBV-related HCC patients (one patient who died from postoperative massive hemorrhage was excluded) revealed that lower lncRNA-6195 expression levels in HCC tissues significantly correlated with a markedly reduced overall survival (OS) (*P* = 0.042, log-rank test; Fig. 1c) of the HBV-related HCC patients. These results together suggest that lncRNA-6195 may play an important role in the pathogenesis and prognosis of HBV-related HCC.

**Table 1:** Relationship between lncRNA-6195 expression and the clinical characteristics of HCC patients

		LncRNA-6195 expression levels		*P* value
		Low expression	High expression	
All cases		23	24	
Age (years)				
≤55		18	17	0.559
>55		5	7	
Gender				
Male		22	23	0.975
Female		1	1	
HBV-DNA (copies/ml)				
≤10^3^		18	13	0.081
>10^3^		5	11	
Tumor size (cm)				
≤ 5		11	10	0.671
> 5		12	14	
Tumors (*n*)				
Multiple		4	6	0.524
Solitary		19	18	
Cirrhosis				
Yes		15	19	0.285
No		8	5	
AFP (ng/ml)				
≤20		6	7	0.813
>20		17	17	
Edmondson grade				
I–II		16	23	0.017*
III–IV		7	1	
Vascular invasion				
Yes		7	11	0.278
No		16	13	
Capsular invasion				
Yes		12	8	0.192
No		11	16	

The median expression level of lncRNA-6195 was used as the cutoff. “Low expression” group include 23 patients whose lncRNA-6195 expression was lower than the median. “High expression” group include 24 patients whose lncRNA-6195 expression was higher than the median. Pearson’s *χ* test and Fisher's exact test were used to analyze the correlation between lncRNA-6195 expression levels and clinical features. *P < 0 .05.

### LncRNA-6195 inhibits cell proliferation in vitro

**Fig. 2:**
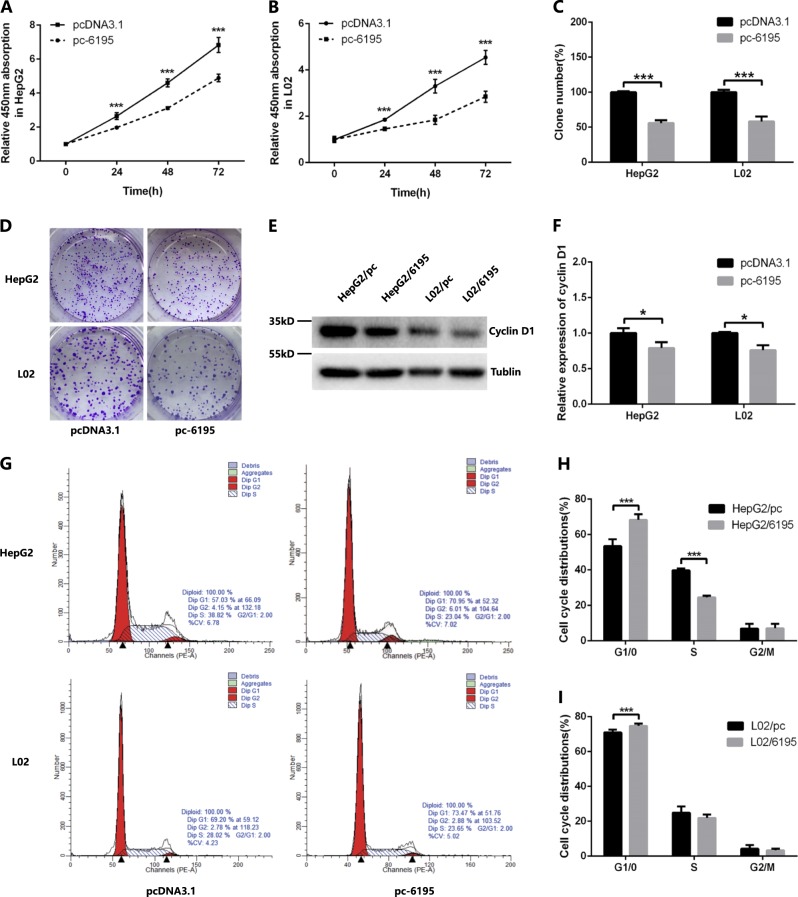
Overexpression of lncRNA-6195 inhibits cell proliferation in vitro. **a**, **b** Overexpression of lncRNA-6195 inhibits cell proliferation in HepG2 and L02 cell lines as assessed by the CCK-8 assay. **c**, **d** Overexpression of lncRNA-6195 inhibits cell proliferation in HepG2 and L02 cell lines as assessed by the colony formation assay. **e**, **f** Cyclin D1 protein is downregulated in lncRNA-6195 overexpressed HepG2 and L02 cell lines. **g**–**i** Overexpression of lncRNA-6195 increases the cell numbers in the G1 phase and inhibits cell cycle progression in HepG2 and L02 cells. (*P < 0.05, ***P < 0.001, Student’s *t* test. Data are represented as mean ± SD.)

To determine its function, the effects of lncRNA-6195 on cell proliferation, apoptosis, migration, and invasion were investigated by gain- and loss-of-function studies in the L02, HepG2, and Huh7 cell lines. The results showed that overexpression of cellular lncRNA-6195 not only suppressed the cell proliferation (Fig. 2a–d) but also inhibited the migration and invasion activities of liver cancer cells, compared with those in the negative control (Supplementary Fig. S1B–E). However, lncRNA-6195 upregulation had no significant effect on cell apoptosis in vitro (Supplementary Fig. S1F).

The L02 and HepG2 cell lines stably overexpressing lncRNA-6195 were constructed using the pc-6195 plasmid and were verified by RT-PCR (Supplementary Fig. S1A), the CCK-8 assay (Fig. 2a, b), and the colony formation assay (Fig. 2c, d). The results showed that overexpression of lncRNA-6195 could suppress the cell proliferation. Subsequently, cell cycle distribution was investigated by flow cytometry, and the data demonstrated that upregulation of lncRNA-6195 increased the proportion of cells in the G1 phase and inhibited the cell cycle progression (Fig. 2g–i). In addition, the cyclin D1 protein was downregulated in the lncRNA-6195-overexpressing L02 and HepG2 cell lines (Fig. 2e, f).

**Fig. 3:**
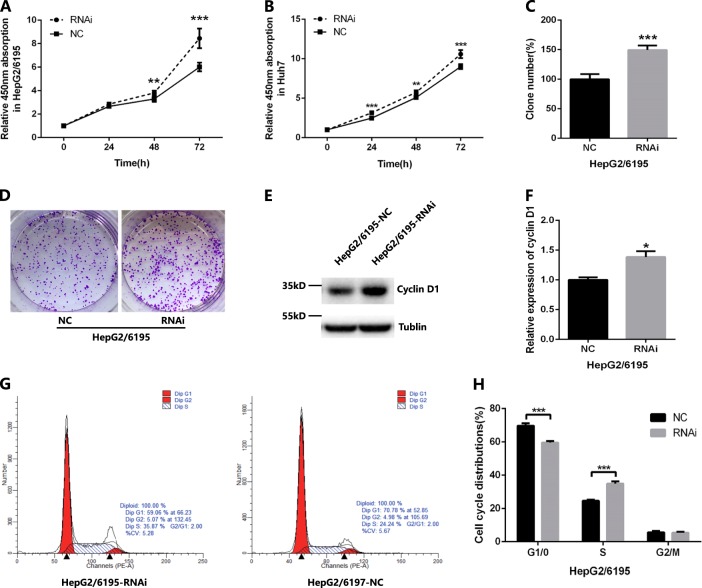
Inhibition of lncRNA-6195 promotes cell proliferation in vitro. **a**, **b** lncRNA-6195 shRNA promoted cell proliferation as assessed by the CCK-8 assay after stable transfection of lncRNA-6195 shRNA(RNAi) or negative control shRNA (NC) in HepG2/6195 (**a**) and Huh7 cells (**b**). **c**, **d** Inhibition of lncRNA-6195 promotes cell proliferation in HepG2/6195 cells as assessed by the colony formation assay. **e**, **f** Western blot results showed that Cyclin D1 protein is upregulated in lncRNA-6195 knock-downed HepG2/6195 cell lines. **g**, **h** Inhibition of lncRNA-6195 decreases the cell numbers in the G1 phase and promotes cell cycle progression as analyzed using flow cytometry. (*P < 0.05, **P < 0.01,***P < 0.001, Student’s *t* test. Data are represented as mean ± SD.)

To further confirm these observations, rescue experiments were performed. Knockdown of the expression of lncRNA-6195 (Supplementary Fig. S2A) significantly promoted the cell proliferation of HepG2/6195 (HepG2 cells stably transfected with pc-6195) and Huh7/6195 (Huh7 cells stably transfected with pc-6195) cells (Fig. 3a–d). In addition, inhibition of the lncRNA-6195 expression upregulated the cyclin D1 protein (Fig. 3e, f) and decreased the proportion of HepG2/6195 cells in the G1 phase (Fig. 3g, h). All these results suggest that lncRNA-6195 may inhibit the cell proliferation in vitro.

### LncRNA-6195 inhibits tumorigenesis in vivo

**Fig. 4:**
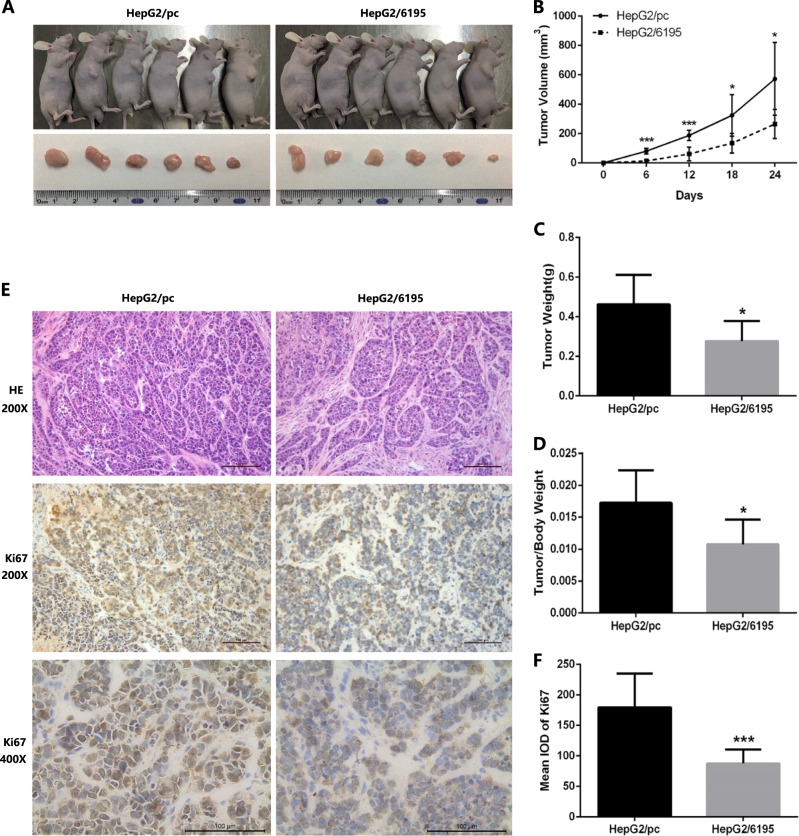
lncRNA-6195 inhibits tumorigenesis of HepG2 cells in vivo. **a** HepG2 cells stably transfected with pcDNA3.1-6195 or pcDNA3.1 vectors were implanted subcutaneously into nude mice (*n* = 6), respectively. These photos show the tumor xenografts 24 days after inoculation. LncRNA-6195-overexpressed HepG2 cells attenuate tumor growth in vivo. **b** The volumes of the subcutaneous tumors which were measured every 6 days after implantation. **c**, **d** The tumor weight and tumor weight/body weight ratio of two groups. **e** Subcutaneous tumors tissue sections were subjected to HE stain (magnification: ×200) or Ki-67 IHC analysis (magnification: ×200; ×400). **f** Assessment of the Ki-67 protein expression in two groups shows that subcutaneous tumors of LncRNA-6195 upregulated group express lower Ki-67 than the control group. (*P < 0.05, ***P < 0.001. Student’s *t* test. Data are shown as mean ± SD.)

The results obtained in BALB/C nude mice, which were subcutaneously injected with HepG2/6195 or HepG2/pc cells, showed that compared with those in the control group, subcutaneous tumors were significantly smaller in the mice injected with HepG2/6195 cells (Fig. 4a–d). Ki-67 immunohistochemistry (IHC) staining revealed lower Ki-67 expression levels in the tumors from the HepG2/6195 group compared with those in the tumors from the control group (Fig. 4e, middle and lower panels, and Fig. 4f), which indicated a slower growth of the HepG2/6195-derived tumors. However, hematoxylin and eosin (HE) staining of tumors from the two groups did not show any significant difference (Fig. 4e, upper panel).

### LncRNA-6195 can combine with ENO1 protein

**Fig. 5:**
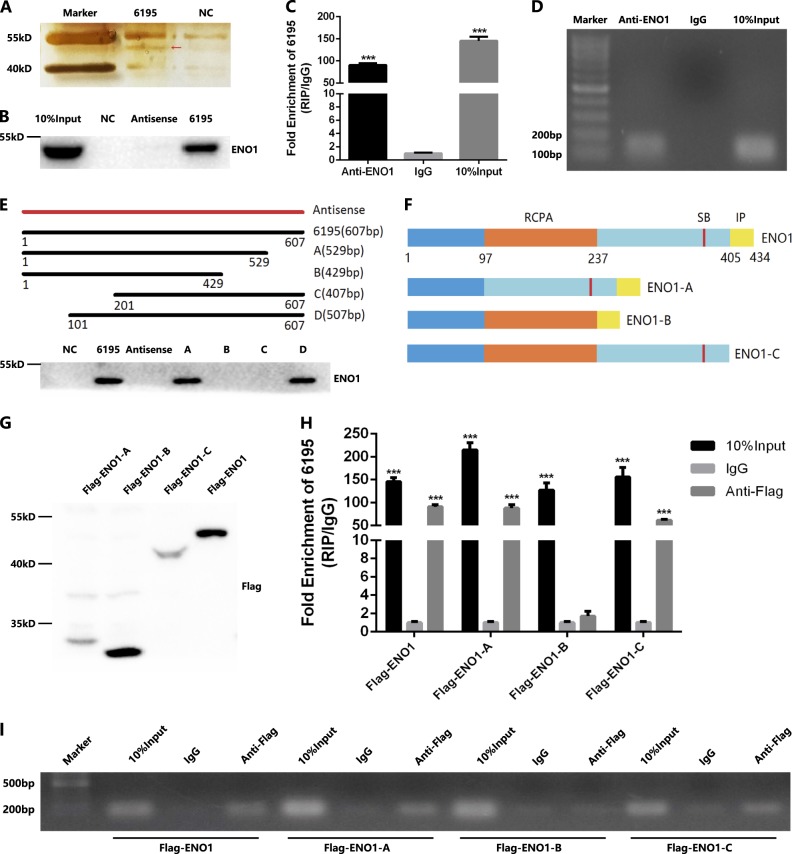
LncRNA-6195 combining with protein ENO1. **a** Silver-stained SDS-PAGE gel-containing proteins derived from RNA pulldown by lncRNA-6195 and negative control RNA. The red arrow indicates the gel cutting for mass spectrometric analysis. **b** Western blot analysis of ENO1 derived from RNA pulldown by lncRNA-6195, its antisense RNA and negative control RNA. Input is the total protein used for RNA pulldown. **c** RT-PCR analysis of RNAs derived from RIP assays in HepG2 cells. **d** Agarose electrophoresis of the PCR products. **e** Western blot analysis of ENO1 derived from RNA pulldown by negative control RNA, lncRNA-6195, its antisense RNA, and different fragments. **f** Graphic illustration of ENO1 and different truncation mutants(RCPA: 97–237aa, region required for repression of c-myc promoter activity; SB: 370–373aa, region required for substrate binding; IP: 405–434aa, region required for interaction with plasminogen). **g** Western blot analysis of Flag-tagged ENO1 and its mutants. **h** RIP experiments were performed using Flag antibody on extracts from HepG2 cells transfected with Flag-tagged full-length or mutant ENO1 expression vectors. The purified RNAs were analyzed by RT-PCR. **i** Agarose electrophoresis of the PCR products. (***P < 0.001. Student’s *t* test. Data are presented as mean ± SD.)

To investigate whether lncRNA-6195 functions by interacting with a specific target, we performed an RNA pull-down assay to identify proteins associated with lncRNA-6195 (Fig. 5a). The precipitated proteins were separated by 12% sodium dodecyl sulfate-polyacrylamide gel electrophoresis (SDS-PAGE) and then silver-stained. The differentially expressed bands, compared with those in the negative control, were excised to be identified by mass spectrometry (Table 2). The results of mass spectrometry indicated that ENO1 might be specifically associated with lncRNA-6195, and subsequent western blot analysis confirmed these results (Fig. 5b). To further verify the association between lncRNA-6195 and ENO1, we performed a RNA immunoprecipitation (RIP) assay with an antibody against ENO1 and a nonspecific antibody (immunoglobulin G (IgG)) using HepG2 cellular extracts. Consistently, we observed a significantly higher enrichment level of lncRNA-6195 with the ENO1 antibody than that with IgG (Fig. 5c, d). Thus, the results of both assays demonstrated that lncRNA-6195 could combine with ENO1 in vitro.

**Table 2:** Mass spectrometry analysis of the proteins pulled down by lncRNA-6195

Protein hits	Mass	Score	emPAI
α-Enolase	47,481	182	0.76
Elongation factor 1-α1	504,51	43	0.07
Keratin, type II cytoskeletal 5	62,568	47	0.06
Transketolase	68,519	31	0.04
Heat-shock cognate 71 kDa protein	71,082	32	0.04
Peroxisome biogenesis factor 1	143,804	37	0.02

*emPAI* exponentially modified protein abundance index

To identify the binding sites of lncRNA-6195, we constructed a series of deletion mutants of lncRNA-6195 and performed an RNA pull-down assay to map the ENO1-binding region. The deletion mutants included the following fragments of lncRNA-6195: A (nucleotides 1–529), B (nucleotides 1–429), C (nucleotides 201–607), and D (nucleotides 101–607). The results indicated that ENO1 interacted with the region between nucleotides 101 and 529 of lncRNA-6195 (Fig. 5e).

We also constructed a series of Flag-tagged deletion mutants of ENO1 and performed a RIP assay to map the lncRNA-6195-binding domain. The deletion mutants included ENO1-A, in which the 97–237-amino acid (aa) region, required for the repression of the c-myc promoter activity, was deleted; ENO1-B, in which the 237–405-aa region between the cleavage sites of ENO1-A and ENO1-C, containing the region (370–373 aa) required for substrate binding, was deleted; and ENO1-C, in which the 405–434-aa region, required for the interaction with plasminogen, was deleted (Fig. 5f, g). The assay data demonstrated the binding of lncRNA-6195 to the 237–405-aa region of ENO1 (Fig. 5h, i), which suggested that lncRNA-6195 might function by regulating the enzyme activity of ENO1.

### LncRNA-6195 can inhibit the enzymatic activity of ENO1

**Fig. 6:**
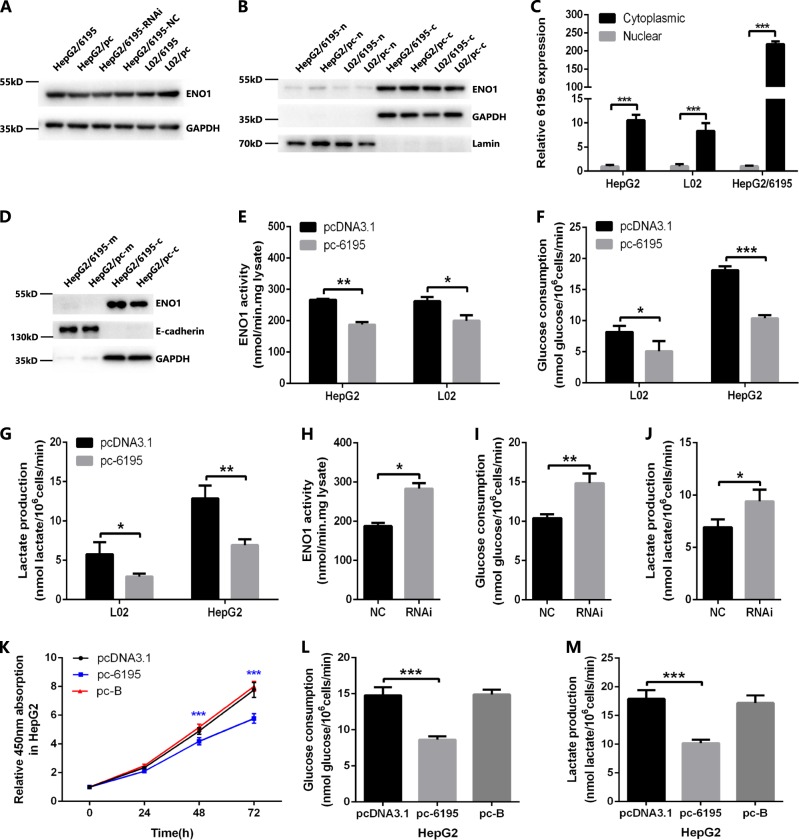
LncRNA-6195 represses HCC progression by inhibiting enzymatic activity of ENO1 **a** The expression level of ENO1 protein has no significant change when lncRNA-6195 was up/downregulated in cells. **b** ENO1 in HepG2 and L02 cells is mainly located in the cytoplasm (-c), and little is found in the nucleus (-n). The location and expression have no significant changes in lncRNA-6195 upregulated cells. **c** LncRNA-6195 is mainly located in the cytoplasm in HepG2, L02, and HepG2/6195 cells. **d** There is almost no ENO1 located on the cell membrane (-m) of HepG2 cells. **e** Enzyme activities of ENO1 is reduced in lncRNA-6195 overexpressed L02 and HepG2 cells. **f**, **g** Glucose consumption and lactate production are both reduced in lncRNA-6195-overexpressed L02 and HepG2 cells. **h** Enzyme activities of ENO1 is promoted in lncRNA-6195 downregulated HepG2/6195 cells. **i**, **j** Glucose consumption and lactate production are both increased in lncRNA-6195 downregulated HepG2/6195 cells. **k** Unlike pc-6195 which inhibits cell proliferation, pc-B has no significant effect on cell proliferation as assessed by the CCK-8 assay after transfected in HepG2 cells. **l**, **m** Compared with the control group (HepG2/ pc), glucose consumption and lactate production have no significant changes in lncRNA-6195 deletion mutant B-overexpressed HepG2 cells. (*P < 0.05, **P < 0.01, ***P < 0.001. Student’s *t* test. Data are represented as mean ± SD.)

Recent studies have demonstrated that lncRNA can interact with proteins through several modes of action, such as the modulation of protein function, regulation of protein–protein interactions, and direct localization within cellular compartments^26^. ENO1 is a multifunctional protein; it plays a role as a key glycolytic enzyme in the cytoplasm while serving as a plasminogen receptor on the surface of cells^14^. To investigate the molecular function of the lncRNA-6195–ENO1 ribonucleoprotein (RNP), the protein expression levels of ENO1 were measured by western blotting in different cell lines. The results showed no significant changes in the expression levels of ENO1 when lncRNA-6195 was upregulated in HepG2 and L02 cells or downregulated in HepG2/6195 cells, compared with those in their corresponding control groups (Fig. 6a). Subsequently, the distribution of ENO1 and lncRNA-6195 was studied in cells. Both ENO1 and lncRNA-6195 were mainly located in the cytoplasm, with little detected in the nucleus in HepG2 and L02 cells (Fig. 6b, c), and almost no ENO1 was detected in the cell membrane in HepG2 cells. Additionally, the localization and expression levels did not significantly change in lncRNA-6195-upregulated cells (Fig. 6d). Lastly, the enzyme activity of ENO1, as well as glucose consumption and lactate production were determined. The data showed that the enzyme activity of ENO1 was reduced in lncRNA-6195-overexpressing L02 and HepG2 cells (Fig. 6e) and increased in lncRNA-6195-downregulated HepG2/6195 cells (Fig. 6h). Consistently, the glucose consumption and lactate production were both reduced in lncRNA-6195-upregulated L02 and HepG2 cells (Fig. 6f, g) and increased in lncRNA-6195-downregulated HepG2/6195 cells (Fig. 6i, j).

To further confirm that the RNA–protein interaction between lncRNA-6195 and ENO1 is the key antitumor function of lncRNA-6195, its deletion mutant B (Fig. 5e) was overexpressed in HepG2 cells transfected with the pc-B plasmid (Supplementary Fig. S3C, D). The results showed that the pc-B transfection had no significant effect on the proliferation of HepG2 cells, as assessed by the CCK-8 assay (Fig. 6k), and there were no significant changes in the glucose consumption and lactate production in lncRNA-6195 deletion mutant B-overexpressing HepG2 cells (Fig. 6l, m).

Based on the above results, we suggest that lncRNA-6195 can repress the HCC progression by combining with ENO1 and inhibiting its enzymatic activity.

## Discussion

HCC is a worldwide disease with a very low 5-year survival rate^1^. There are numerous alterations in DNA, RNA, and proteins that enable cancer cells to immortally replicate, resist cell death, invade, and metastasize^27^. Recently, emerging evidence has greatly advanced our understanding of essential roles of lncRNAs in the pathogenesis and progression of HCC^28–30^. Researchers have found that HBx can alter the expression of lncRNAs, many of which have been suggested to be closely related to HCC^11–13^. Although hundreds of lncRNAs have been functionally characterized, the vast majority still remain to be studied. Our previous study has found that lncRNAs were aberrantly expressed in HBx-overexpressing L02 cells. Expression of lncRNA-6195 was one of the most significantly altered in LO2/HBx cells. In this study, we demonstrated that lncRNA-6195, whose function had never been investigated before, could inhibit the HCC growth in vitro and in vivo.

Recent studies have shown that lncRNAs can regulate the HCC growth in a variety of ways. In particular, lncRNAs induce many important cancer phenotypes through their interactions with other cellular macromolecules, including DNA, proteins, and RNA^26^. After an unsuccessful search of all commonly used databases in an attempt to predict potential nucleotide sequences that could combine with lncRNA-6195, we speculated that lncRNA-6195 might repress the HCC growth by interacting with proteins. The RNA pull-down assay and mass spectrometry revealed that lncRNA-6195 could combine with the ENO1 protein. ENO1 is a multifunctional protein that has been implicated in several critical biological progresses in cancer, including the proliferation, migration, and invasion. In the cytoplasm, ENO1 catalyzes not only the transformation of 2PG to PEP during glycolysis but also the reverse conversion of PEP to 2PG during glycogen synthesis. By participating in anaerobic glycolysis (Warburg effect) and providing ATP, ENO1 is thought to promote cancer development and progression^31^. On the cell surface, ENO1 binding to plasminogen results in enhanced plasminogen activation, localization of the plasmin proteolytic activity on the cell surface, and protection of plasmin from α-2-antiplasmin^32,33^. The plasminogen activation system is involved in cancer cell invasion and metastasis^34–36^. Recent studies have demonstrated that ENO1 is overexpressed in HCC tissue, which is correlated with the degree of tumor differentiation and progression^18,37^. The knockdown of ENO1 expression with a small interfering RNA significantly inhibited the proliferation of an HCC cell line^17^, whereas upregulation of the activity of the glycolytic enzyme ENO1 could promote the occurrence and development of cancer^38^.

As we found in this study, lncRNA-6195 did not affect the expression level of ENO1, and both lncRNA-6195 and ENO1 were mainly located in the cytoplasm. Their intracellular distribution suggested that the mechanism of action of the lncRNA-6195–ENO1 RNP might be associated with the enzyme activity. The data demonstrated that lncRNA-6195 could reduce the enzyme activity of ENO1. Further research led to the identification of the binding domain of ENO1 as the 237–405-aa region, which contains the substrate-binding site. This finding implies that lncRNA-6195 may repress the enzyme activity of ENO1 through the disruption of ENO1–substrate interaction.

**Fig. 7:**
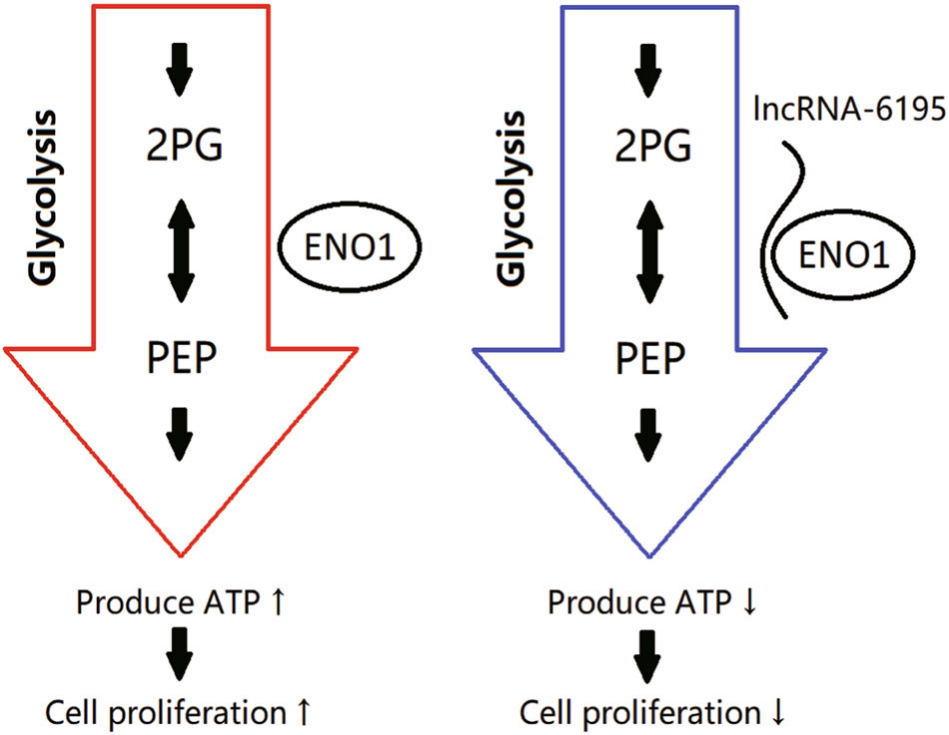
A model for the regulatory mechanisms of lncRNA-6195 in HCC. The big red arrow represent one enzyme-catalyzed reaction of glycolysis that was catalyzed by ENO1 and release high-energy molecules ATP and NADH to promote cell proliferation. The big blue arrow represent that lncRNA-6195 can inhibit cell proliferation through binding with ENO1 and repressing its enzyme activity by disturbing the combination between ENO1 and its substrate

Collectively, our results reveal that enhanced expression of lncRNA-6195 can reverse the progression of HCC via lncRNA-6195 combining with ENO1 and inhibiting its enzymatic activity (Fig. 7). These findings suggest new therapeutic strategies for the prevention and treatment of HCC.

## Materials and methods

### Patient samples

In this study, HBV-related HCC specimens and adjacent non-tumorous liver tissues were collected from 47 patients who were pathologically diagnosed with HBV-related HCC between July 2014 and April 2015 at the XiangYa Hospital, Central South University (Changsha, China). Patients who received radiotherapy, embolotherapy, or chemotherapy were excluded. All patients were followed up for 24 months post surgery. OS was defined as the interval between tumor resection and death or the last follow-up examination. The patients’ clinicopathological characteristics are summarized in Supplementary Table S1. The study was approved by the Ethics Committee of the XiangYa Hospital, and written informed consent was obtained from all patients.

### RNA isolation andRT-PCR

Total RNA was extracted from HCC and adjacent liver tissues or cultured cells using the EZNA total RNA kit I (Omega, USA), while nuclear and cytoplasmic RNA was extracted from cultured cells using the PARIS kit (Life Technologies, USA) according to the manufacturer’s instructions. Subsequently, RNA was reverse-transcribed into cDNA using the PrimeScript RT reagent kit with gDNA Eraser (Takara, Japan). RNA expression levels were measured by qRT-PCR on the ABI 7500 Fast real-time PCR platform using SYBR® Premix Ex Taq II (Takara, Japan). Relative quantification was performed using the 2^−ΔΔCt^ method. The primers used are listed in Supplementary Table S2.

### Cell lines

The Huh7, HepG2, 293T, and L02 cell lines were purchased from the Cell Bank of the Chinese Academy of Sciences (Shanghai, China). Huh7, HepG2, and 293T cells were maintained in Dulbecco’s modified Eagle’s medium (Gibco, USA) supplemented with 10% fetal bovine serum (FBS; Gibco, USA). L02 cells were maintained in RPMI 1640 medium (Gibco, USA) supplemented with 10% FBS. All cells were incubated at 37 °C in an incubator with 5% CO_2_.

### Plasmid and lentiviral constructions

The lncRNA-6195 expression plasmid vector (pc-6195) and the lncRNA-6195 part B expression plasmid vector (pc-B) were constructed by inserting a full-length lncRNA-6195 or a part B lncRNA-6195 fragment (Supplementary Fig. S3B) into the *Bam*HI/*Eco*RI site of pcDNA3.1. The flag-tagged full-length ENO1 protein and ENO1 deletion mutants were constructed by inserting a full-length *ENO1* or fragments of *ENO1*-A/B/C mutants (Fig. 5f) into the *Bam*HI/*Eco*RI site of pcDNA3.1. Short hairpin RNAs (shRNAs) against lncRNA-6195 were designed (Gene Pharma, China) and cloned into the *Bam*HI/*Eco*RI sites of Lenti-X expression vectors (Clontech, USA). Recombinant pLVX-shRNA vectors and the Lenti-X HTX packaging system (Clontech, USA) were used to produce high-titer lentivirus in 293T packaging cells. The sequences are described in Supplementary Table S2.

### Cell transfection and RNA interference

The lncRNA-6195 expression vector, control plasmid vector, and flag-tagged full-length or mutant ENO1 expression vectors were transfected into cells using the Fugene 6 reagent (Promega, USA) according to the manufacturer’s instructions. The expressing cells were established using G418. The lncRNA-6195 shRNA or control shRNA was transfected into cells using polybrene (Genomeditech, China). Stably transfected cell lines were selected using puromycin, and the expression level of lncRNA-6195 was confirmed by qRT-PCR.

### Cell proliferation assay

Cells were seeded in 96-well plates at a density of 5000 cells per well in 100 μL of complete medium. At the indicated time points, 10 μL of the Cell Counting Kit-8 (CCK-8) reagent (Beyotime, China) was added to each well, and the plate was incubated at 37 °C for an additional 1 h. The absorbance in each well was measured at 450 nm using a microplate reader.

### Colony formation assay

Cells were seeded in 6-well plates at a density of 500 cells per well. After approximately 2 weeks, the culture medium was removed, cells were stained with crystal violet, and colonies that had more than 50 cells were counted.

### Cell cycle and cell apoptosis assay

The cell cycle and the cell apoptosis were measured on flow cytometer (FACSCalibur, BD Biosciences, San Jose, CA) platform using Cell Cycle Analysis kit (Beyotime, China) or Annexin V-FITC Apoptosis Detection kit (KeyGen Biotech, China). All data were analyzed by the FlowJo X software.

### Proteins isolation and western blot analysis

Total proteins were extracted from cultured cells using cell lysis buffer for western and IP (Beyotime). Membrane proteins and nuclear and cytoplasmic proteins were extracted from cultured cells using a Mem-PER Plus membrane protein extraction kit (Thermo Scientific, USA) and NE-PER nuclear and cytoplasmic extraction reagents (Thermo Scientific), respectively, according to the manufacturer’s instructions. Proteins were separated by 8–12% SDS-PAGE and transferred to polyvinylidene difluoride membranes (0.25 μm; Millipore). The membranes were blocked with 5% bovine serum albumin in Tris-buffered saline with Tween-20 (TBST) for 1 h, then washed with TBST, and incubated with primary antibodies overnight at 4 °C. After subsequent incubation with horseradish peroxidase-conjugated secondary antibodies for 1 h at 25 °C, signals were visualized using an enhanced chemiluminescence method (Bio-Rad). The relative band intensity was measured using the Image Lab software. Anti-tubulin and glyceraldehyde 3-phosphate dehydrogenase antibodies were obtained from Auragene Bioscience (Changsha, China). The flag antibody was obtained from Sigma-Aldrich (St. Louis, MO, USA). Other antibodies used were obtained from Abcam (Cambridge, MA, USA).

### Animal experiments

BALB/C nude mice (male, 4-week-old) were purchased from the Hunan Technology Transfer Center Experimental Animal Division of the Chinese Academy of Sciences (Hunan, China). All animal experiments were conducted in compliance with the regulations of the Institutional Animal Care and Use Committee of the Department of Laboratory Animals of CSU in China. To investigate the effects of lncRNA-6195 on tumorigenesis in vivo, we subcutaneously injected HepG2/6195 or HepG2/pc cells (stably transfected with pc-6195 or pcDNA3.1, respectively) into BALB/C nude mice (*n* = 6). The volumes of subcutaneous tumors were measured every 6 days after implantation. After 24 days, the mice were sacrificed to harvest tumor tissue for analysis. Tissue sections were stained with HE or subjected to Ki-67 immunohistochemical staining.

### Immunohistochemistry

IHC for Ki-67 was performed on paraffin-embedded tumor sections using a primary antibody against Ki-67 (Abcam, USA) and a goat anti-rabbit IgG horseradish peroxidase-conjugated secondary antibody (Abcam, USA). Proteins were visualized in situ with 3,3′-diaminobenzidine, and photographs were analyzed using the Image-Pro Plus 6.0 software.

### RNA pull-down assay

LncRNA-6195, its A–D fragments, and antisense RNA were transcribed from the pcDNA3.1-6195 vector using the TranscriptAid T7 high-yield transcription kit. RNA–protein pull-down experiments were performed using the Pierce™ magnetic RNA–protein pull-down kit according to the manufacturer’s instructions. Retrieved proteins were separated by 12% SDS-PAGE, then silver-stained using the Pierce^®^ silver stain kit, and specific bands were cut out and analyzed by mass spectrometry using the Mascot software. All the kits used in this assay were obtained from Thermo Fisher Scientific (Waltham, MA, USA).

### RIP assay

RIP experiments were performed using the Magna RIP™ RNA-binding protein immunoprecipitation kit (Millipore, Danvers, MA, USA) according to the manufacturer’s instructions. The anti-ENO1 antibody for the RIP assay was obtained from Abcam. RT-PCR was used to examine whether co-precipitated RNA contained lncRNA-6195. The primers used are presented in Supplementary Table S2.

### Glucose consumption and lactate production

Cells were seeded in 6-well plates at a density of 5 × 10^5^ cells per well. After 24 h of incubation, the medium was collected and centrifuged at 2000 rpm for 5 min to remove cell debris. Glucose and lactate levels were determined in the culture medium using an automatic biochemical analyzer (Beckman, Germany). Cell-free medium was used as the blank control.

### Enzyme activity assay

The enzymatic activity of ENO1 was measured in different cell lines using an enolase activity assay kit (Sigma-Aldrich, St Louis, MO, USA) according to the manufacturer’s instructions.

### Statistical analysis

Statistical analysis was performed using the SPSS 24.0 and GraphPad Prism 6 software. Each experiment was performed at least three times. The expression of lncRNA-6195 and HBx in patient HCC tissue and tumor-adjacent tissue was compared by a paired sample *t* test. Survival curves were calculated using the Kaplan–Meier method and a log-rank test. The *χ*^2^ test, Fisher’s exact test, and Student’s *t* test were used for comparison between groups. Normally distributed data are expressed as the mean ± standard deviation (SD). All *P* -values were two-sided, and *P* < 0.05 was accepted as statistically significant.

## Electronic supplementary material

Supplementary Figure Legends

Supplementary Table 1

Supplementary Table 2

Supplementary Figure 1

Supplementary Figure 2

Supplementary Figure 3
