# Supplementary material for: A novel lncRNA, TCONS_00006195, represses hepatocellular carcinoma progression by inhibiting enzymatic activity of ENO1
Source: Cell Death Dis. 2018 Dec 5;9(12):1184. doi: 10.1038/s41419-018-1231-4 (PMC6281672; doi:10.1038/s41419-018-1231-4)
Supplement: Supplementary file 1 — Supplementary Figure Legends [file 41419_2018_1231_MOESM1_ESM.docx]

**Supplementary Figure Legends**

**Fig.S1. Overexpression of** **lncRNA-6195 represses cell** **migration in vitro.**

(A) The relative expression level of pc-6195 or pcDNA3.1 plasmid’s stable transfected L02 and HepG2 cell lines was measured by RT-PCR. (B-E) The high level of lncRNA-6195 represses cell migration and invasion. (B) The wound-healing assay results showed that overexpression of lncRNA-6195 inhibited the wound-healing ability of HepG2 cells. (C) The percentage of invaded cells was decreased in lncRNA-6195 overexpressed HepG2 cells. (*** P<0.001. Student’s t-test. Data are shown as the mean ± SD.) (D) Cell morphology graph of wound-healing assay. (E) Cell morphology graph of invasive cells from transwell assay with or without Matrigel. (Magnification: x100) (F) lncRNA-6195 overexpression had no significant effect on cell apoptosis in L02 and HepG2 cells as analyzed using flow cytometry.

**Fig.S2.** **Inhibition of lncRNA-6195** **promotes cell migration in vitro.**

(A) The relative expression of lncRNA-6195 in L02, HepG2/6195 and Huh7 cells with pshR-lncRNA-6195 or pshR-NC stable transfection. (B) Inhibition of lncRNA-6195 can promote the wound-healing ability of HepG2/6195 cells as assessed using wound-healing assay. (C) The percentage of invaded cells was increased in lncRNA-6195 inhibited HepG2/6195 cells. (*** P<0.001. Student’s t-test. Data are shown as the mean ± SD.) (D) Cell morphology graph of wound-healing assay. (E) Photograph of invasive cells from transwell assay with or without Matrigel. (Magnification: x100) (F) Low-level expression of lncRNA-6195 had no significant effect on cell apoptosis in L02 and HepG2/6195 cells as analyzed using flow cytometry.

**Fig.S3. Overexpression of lncRNA-6195 and its** **deletion mutant B.**

(A) The negative control of Ki67 protein IHC analysis (Magnification:x400). (B) The transcript location of lncRNA-6195 deletion mutant B and two test parts(P1& P2). (C,D) The relative expression level of P1 or P2 in HepG2 cells transfected with pc-6195, pc-B or pcDNA3.1 was measured by RT-PCR. (E) Nucleotide sequence of the full-length cDNA of human lncRNA-6195 (5’ to 3’).
