# Supplementary material for: A novel lncRNA, TCONS_00006195, represses hepatocellular carcinoma progression by inhibiting enzymatic activity of ENO1
Source: Cell Death Dis. 2018 Dec 5;9(12):1184. doi: 10.1038/s41419-018-1231-4 (PMC6281672; doi:10.1038/s41419-018-1231-4)
Supplement: Supplementary file 2 — Supplementary Table 1 [file 41419_2018_1231_MOESM2_ESM.docx]

**Supporting Materials**

**Supporting Table 1. The clinicopathological characteristics of 47 HCC patients.**

| Varible | n=47 | |
| --- | --- | --- |
|  | Number | Percentage |
| Age(years) |  |  |
| ≤55 | 35 | 74.5% |
| >55 | 12 | 25.5% |
| Sex |  |  |
| Male | 45 | 95.7% |
| Female | 2 | 4.3% |
| Tumor differentiation |  |  |
| I-II | 39 | 83.0% |
| III-IV | 8 | 17.0% |
| TNM stage |  |  |
| I-II | 35 | 74.5% |
| III-IV | 12 | 25.5% |
| Tumor size(cm) |  |  |
| ≤5 | 21 | 44.7% |
| >5 | 26 | 55.3% |
| Tumor number |  |  |
| Solitary | 37 | 78.7% |
| Multiple | 10 | 21.3% |
| AFP(ug/L) |  |  |
| ≤20 | 13 | 27.7% |
| >20 | 34 | 72.3% |
| Encapsulation |  |  |
| No | 27 | 57.4% |
| Complete | 20 | 42.6% |
| Vascular invasion |  |  |
| Absent | 29 | 61.7% |
| Present | 18 | 38.3% |
| Liver cirrhosis |  |  |
| Without | 13 | 27.7% |
| With | 34 | 72.3% |
| HBV-DNA (copies/ml) |  |  |
| ≤10^3^ | 31 | 66.0% |
| >10^3^ | 16 | 34.0% |

TNM, tumor-node-metastasis; AFP, α-fetoprotein;
