# Supplementary material for: A novel lncRNA, TCONS_00006195, represses hepatocellular carcinoma progression by inhibiting enzymatic activity of ENO1
Source: Cell Death Dis. 2018 Dec 5;9(12):1184. doi: 10.1038/s41419-018-1231-4 (PMC6281672; doi:10.1038/s41419-018-1231-4)
Supplement: Supplementary file 3 — Supplementary Table 2 [file 41419_2018_1231_MOESM3_ESM.docx]

**Supporting Materials**

**Supporting Table 2. Sequences of primers and shRNA used in this study.**

| Name | Sequences | |
| --- | --- | --- |
| RT-PCR primers | | |
| LncRNA-6195 | Sense | 5'- GAACACAGGAAAAAAGCAGC-3' |
|  | Anti-sense | 5'- ATGACAAAATACTACAGAATGGG-3' |
| GAPDH | Sense | 5'- CAGGAGGCATTGCTGATGAT-3' |
|  | Anti-sense | 5'- GAAGGCTGGGGCTCATTT-3' |
| shRNA target sequences | | |
| 6195-shRNA | Sense | 5'- GCTGAACATTGAAAGCACT-3' |
|  | Anti-sense | 5'- AGTGCTTTCAATGTTCAGC-3' |
| shRNA NC | Sense | 5'- UUCUCCGAACGUGUCACGUTT -3' |
|  | Anti-sense | 5'- ACGUGACACGUUCGGAGAATT -3' |
| Full long clone primers | | |
| LncRNA-6195 | Sense | 5'- CTTCAGATGTCTATCTGGAGTTCAGG-3' |
|  | Anti-sense | 5'- ATTTTGAATCTGTTTGAAAGCAATC-3' |
| ENO1 | Sense | 5'-ATGTCTATTCTCAAGATCCATGCCAGG-3' |
|  | Anti-sense | 5'- CTACTTGGCCAAGGGGTTTCTGAAG-3' |
| Deletion mutants primers | | |
| LncRNA-6195-A | Sense | 5'- CTTCAGATGTCTATCTGGAGTTCAGG-3' |
|  | Anti-sense | 5'- ACAGTTCCTCTCTGAGCCAAGC-3' |
| LncRNA-6195-B | Sense | 5'- CTTCAGATGTCTATCTGGAGTTCAGG-3' |
|  | Anti-sense | 5'- GGATAGCCCCAGTTTCAGACC-3' |
| LncRNA-6195-C | Sense | 5'- ATTTTGTCATGGCAGCCCAAG-3' |
|  | Anti-sense | 5'- ATTTTGAATCTGTTTGAAAGCAATC-3' |
| LncRNA-6195-D | Sense | 5'- CTCATCAGGAACTAAATCAGTTGGC-3' |
|  | Anti-sense | 5'- ATTTTGAATCTGTTTGAAAGCAATC-3' |
