# Supplementary figures and images for: A novel lncRNA, TCONS_00006195, represses hepatocellular carcinoma progression by inhibiting enzymatic activity of ENO1
Source: Cell Death Dis. 2018 Dec 5;9(12):1184. doi: 10.1038/s41419-018-1231-4 (PMC6281672; doi:10.1038/s41419-018-1231-4)

**A**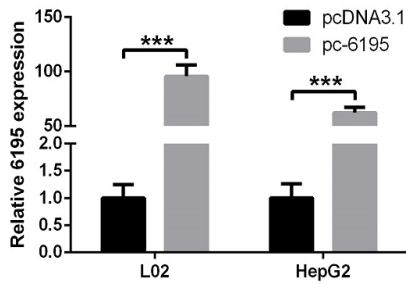**B**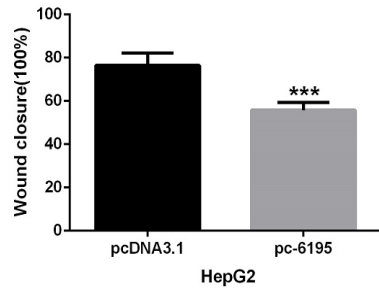**C**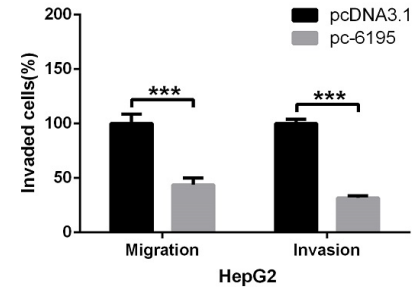**D**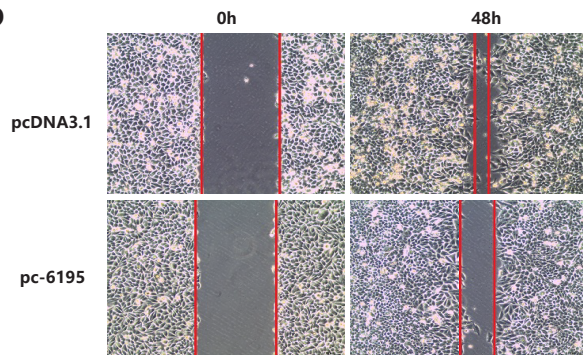**E**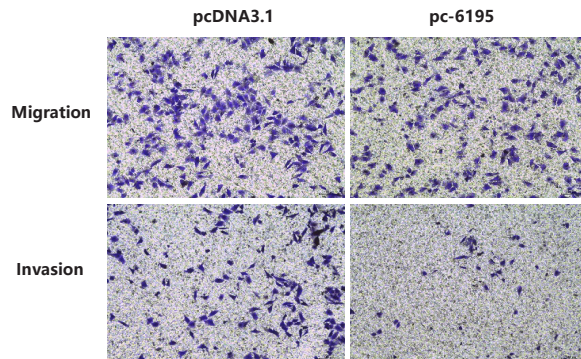**F**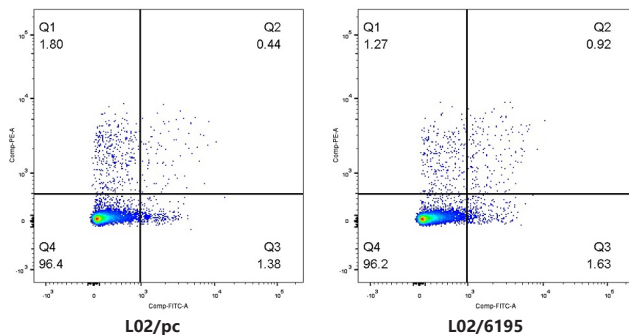**G**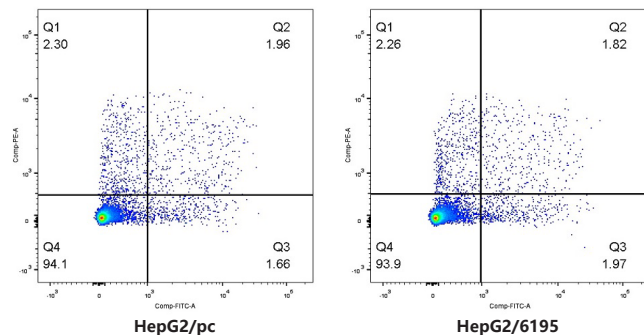

Supplement: Supplementary file 4 — Supplementary Figure 1 [file 41419_2018_1231_MOESM4_ESM.pdf]

**A**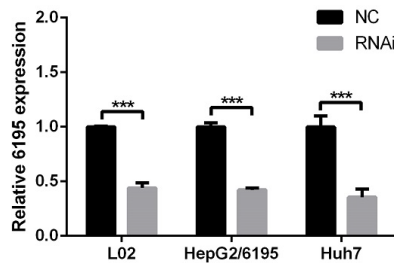**B**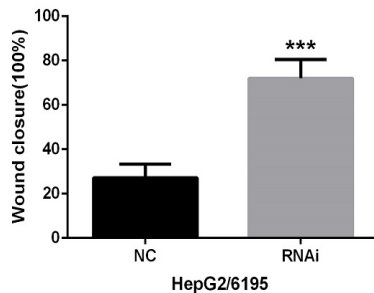**C**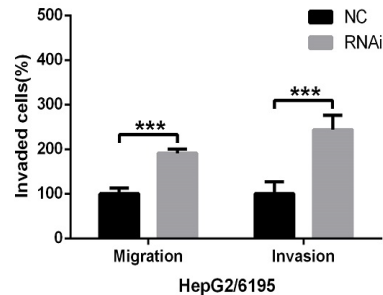**D**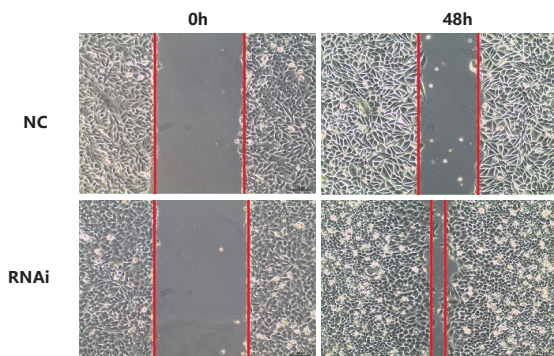**E**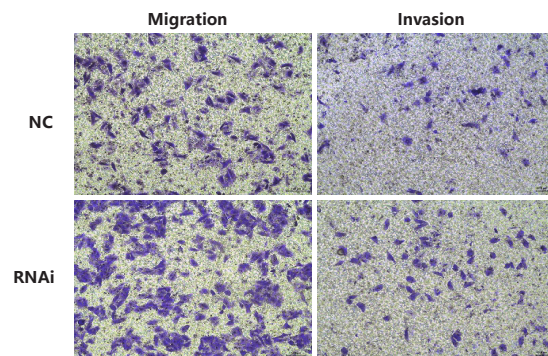**F**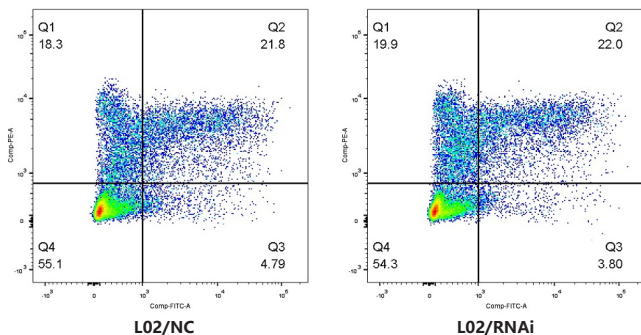**G**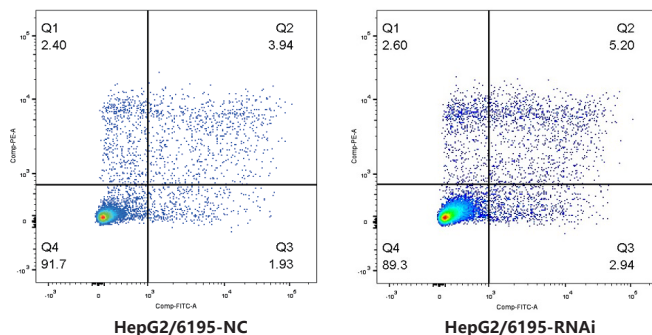

Supplement: Supplementary file 5 — Supplementary Figure 2 [file 41419_2018_1231_MOESM5_ESM.pdf]
