# Supplementary material for: A novel lncRNA, TCONS_00006195, represses hepatocellular carcinoma progression by inhibiting enzymatic activity of ENO1
Source: Cell Death Dis. 2018 Dec 5;9(12):1184. doi: 10.1038/s41419-018-1231-4 (PMC6281672; doi:10.1038/s41419-018-1231-4)
Supplement: Supplementary file 6 — Supplementary Figure 3 [file 41419_2018_1231_MOESM6_ESM.pdf]

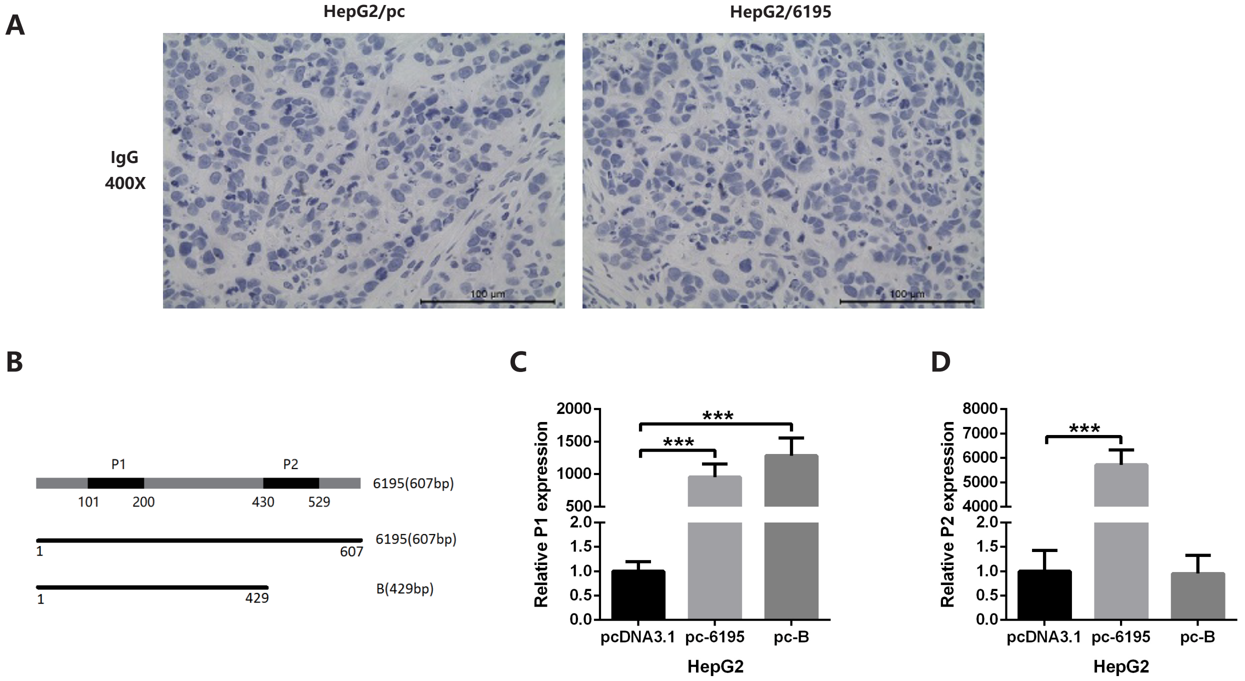

E

```
  1  CTTCAGATGT CTATCTGGAG TTCAGGGTTT CCTGGGTTCT GGTCTGCCCT GAATCAAAAG AACACAGGAA
 71  AAAAGCAGCT GTTTGCGAGG AAGACAGCCC TCATCAGGAA CTAAATCAGT TGGCACCTTG ATCTCAGACT
141  TCCAACCTCC AGAACTGCGA GAAATACATT TCTGTTGTTT AGGCCACCCA TTCTGTAGTA TTTTGTCATG
211  GCAGCCCAAG CTGACTAATA TAACTACCAT TAACAGTCTG AAATTGGGCC AGGGACAGTG GCTCACACAT
281  GTAATCCTAG CACTCTGAGA GGTCAAGGTA GGAGGATACC TTGAGACCAG GTTCAACACC AGCCTGGGCA
351  ACATAGTGAG ACCCACATCT CTATTTAAAA AAAACAAATC AAACAAACAA AAAGAATGGT CTGAAACTGG
421  GGCTATCCAA GCTGAACATT GAAAGCACTA TAGCTACAGC AAAGAAGTAG ACTATTATTT AAGGCAATAT
491  TATGAGTTCA GTCTCTGCTT GGCTCAGAGA GGAACTGTAA ACTCCTCTTT GCCATTGGTG GCCTTTAGGC
561  CACACATCAC TGGTGTTGGC AAGATTGCTT TCAAACAGAT TCAAAAT
```
